# Clinical evaluation of DIAGNOVIR SARS-CoV-2 ultra-rapid antigen test performance compared to PCR-based testing

**DOI:** 10.1038/s41598-023-31177-8

**Published:** 2023-03-17

**Authors:** Ali Aytac Seymen, Ezgi Gulten, Erol Ozgur, Bülend Ortaç, Irem Akdemir, Gule Cinar, Elif Mukime Saricaoglu, Gulen Guney-Esken, Erman Akkus, Fusun Can, Zeynep Ceren Karahan, Alpay Azap, Erkan Tuncay

**Affiliations:** 1 EA Teknoloji LLC Bilkent CyberPark, 06800 Ankara, Turkey; 2 Felisya Biyomedikal, Bilkent, 06800 Ankara, Turkey; 3 Institute of Materials Science and Nanotechnology, National Nanotechnology Research Center (UNAM), Bilkent University, Ankara, 06800 Turkey; 4 Department of Infectious Diseases and Clinical Microbiology, Faculty of Medicine, Ankara University, Ankara, 06230 Turkey; 5 James C. Wyant College of Optical Sciences, University of Arizona, Tucson, AZ 85721 USA; 6 School of Medicine, Department of Medical Microbiology, Koc University, Istanbul, Turkey; 7 Koc University IsBank Research Center for Infectious Diseases (KUISCID), Istanbul, Turkey; 8 Department of Internal Medicine, Faculty of Medicine, Ankara University, Ankara, 06230 Turkey; 9 Department of Medical Microbiology, Faculty of Medicine, Ankara University, Ankara, 06230 Turkey; 10 Departments of Biophysics, Faculty of Medicine, Ankara University, Ankara, 06230 Turkey

**Keywords:** Biophysics, Microbiology

## Abstract

Coronavirus Disease-19 (COVID-19) is a highly contagious infectious disease caused by the severe acute respiratory syndrome coronavirus 2 (SARS-CoV-2). The development of rapid antigen tests has contributed to easing the burden on healthcare and lifting restrictions by detecting infected individuals to help prevent further transmission of the virus. We developed a state-of-art rapid antigen testing system, named DIAGNOVIR, based on immune-fluorescence analysis, which can process and give the results in a minute. In our study, we assessed the performance of the DIAGNOVIR and compared the results with those of the qRT-PCR test. Our results demonstrated that the sensitivity and specificity of the DIAGNOVIR were 94% and 99.2%, respectively, with a 100% sensitivity and 96.97% specificity, among asymptomatic patients. In addition, DIAGNOVIR can detect SARS‑CoV‑2 with 100% sensitivity up to 5 days after symptom onset. We observed that the DIAGNOVIR Rapid Antigen Test’s limit of detection (LoD) was not significantly affected by the SARS‑CoV‑2 variants including Wuhan, alpha (B1.1.7), beta (B.1.351), delta (B.1.617.2) and omicron (B.1.1.529) variants, and LoD was calculated as 8 × 10^2^, 6.81 × 10^1.5^, 3.2 × 10^1.5^, 1 × 10^3^, and 1 × 10^3.5^ TCID50/mL, respectively. Our results indicated that DIAGNOVIR can detect all SARS-CoV-2 variants in just seconds with higher sensitivity and specificity lower testing costs and decreased turnover time.

## Introduction

Coronavirus Disease-19 (COVID-19) is caused by severe acute respiratory syndrome coronavirus-2 (SARS-CoV-2), which emerged in December 2019 in Wuhan, Hubei province, China and rapidly spread all around the world^1^. As of November 2022, there have been more than 630 million have been infected with SARS-CoV-2 and about 6.7 million have died globally from COVID-19 disease^2^.

Cases have started to rise again after the restrictions are lifted following vaccination especially in the USA and Europe in the middle of 2021. According to WHO, more than 10 billion doses of vaccine have been administered in the world. More than half of the population worldwide has received at least one dose of the available vaccines^3^. However, vaccination rates remained low in low to middle-income countries. It also became lower in some high-income countries such as USA due to anti-vaccine groups^4^. After the release of vaccines, the virus was expected to be eradicated in a short time, which is yet to be fulfilled. For that reason, testing for the COVID-19 is still being used as a public health strategy to identify individuals if they are infected with SARS-CoV-2 regardless of whether they are at risk of spreading the infection to others^5,6^.

SARS-CoV-2 is transmitted by exposure to infectious respiratory droplets and its presence is determined by reverse transcription-polymerase chain reaction (RT-PCR) analysis^7^. Although RT-PCR analysis is considered the reference method for COVID-19 diagnosis, it has some limitations, including the accessibility of the patients to the test and the capacity of the laboratories, which can affect the turnaround time. In addition, dedicated and educated professional staff, automated platforms, and specialized laboratory types of equipments are generally required to provide fast and efficient results^8,9^. Most importantly, the PCR test is not rapid and is not cost-effective. In response to the COVID-19 pandemic being declared a public health emergency, test kit manufacturers developed rapid diagnostic devices that detect the presence of SARS-CoV-2 antigens in patient samples^10^. The development of rapid antigen tests has contributed to easing the burden on healthcare and lifting restrictions. In contrast to RT-PCR tests, rapid antigen tests require less laboratory capacity and skilled staff, can be applied as a point-of-care (POC) tests and are also more affordable^11^. These tests can be performed by trained healthcare staff, and even some of them can be applied by the patients. Most importantly test results are delivered within minutes. Therefore, isolation of individuals with SARS-CoV-2 infection with early diagnosis is facilitated^11^.

Here, we present a newly developed SARS-CoV-2 rapid antigen testing system, named DIAGNOVIR, based on immune-fluorescence analysis. In summary, the system is composed of a single used pathogen detection chip, which contains fluorescence-labeled antibodies with a mixture of fluorophores as a donor and acceptor pair in Forster’s Resonance Energy Transfer (FRET), both targeted to the spike proteins of the SARS-CoV-2, and a fluorescence detector.

This study aimed to evaluate the performance of the DIAGNOVIR system for rapid detection of SARS-CoV-2 using nasopharyngeal swab specimens from patients admitted to the infectious diseases department with or without symptoms of COVID-19.

## Results

### Comparison of RT-PCR and the DIAGNOVIR antigen test

Between 29th March 2021 and 12th May 2021, 420 volunteers were included in the study. Due to inadequate sample collection, nine samples were excluded. Of the remaining 411 participants, 50.8% (n = 209) were male. SARS-CoV-2 RT-PCR test was positive in 166 (40.4%) patients. Of the SARS-CoV-2 RT-PCR-positive samples for which threshold cycle (Ct) values were determined as < 26, 26–30 and 31–35, the DIAGNOVIR test was positive in 98.6% (72/73), 90.1% (50/55) and 89.5% (34/38), respectively (Table 1). No statistically significant difference was found between different Ct groups, indicating a high performance of DIAGNOVIR even in patients with low viral load (p = 0.119). SARS-CoV-2 RT-PCR and DIAGNOVIR test results are presented in Table 1 and the supplementary document (Supplementary Table 1).

**Table 1:** SARS-CoV-2 RT-PCR and DIAGNOVIR test results.

	All patients, n (%)		SARS-CoV-2 RT-PCR positive samples stratified through Ct values, n (%)			P value
	RT-PCR negative	RT-PCR positive	< 26	26–30	31–35	
DIAGNOVIR negative	243 (99.2)	10 (6)	1 (1.4)	5 (9.1)	4 (10.5)	0.119
DIAGNOVIR positive	2 (0.8)	156 (94)	72 (98.6)	50 (90.1)	34 (89.5)	
Total	245 (100)	166 (100)	73 (100)	55 (100)	38 (100)	

The sensitivity and specificity of DIAGNOVIR in the whole study population were 94% (95% CI 89.8–97.2%) and 99.2% (95% CI 97.08–99.9%), respectively. The overall positive percent agreement (PPA), negative percent agreement (NPA), positive likelihood ratio (PLR), negative likelihood ratio (NLR), the area under the curve (AUC), and accuracy were 98.7% (%95.2–%99.7), 96.1% (93–97.8%), 115.12 (28.94–457.96), 0.061 (0.033–0.111), 0.966 (0.94–0.98) and 97.08% (94,96–98,48%), respectively. The diagnostic performance of DIAGNOVIR is summarized in Table 2.

**Table 2:** Diagnostic performance of DIAGNOVIR.

DIAGNOVIR performance	All patients
Sensitivity (95% CI)	94% (89.2–97.1%)
Specificity (95% CI)	99.2% (97.1–99.9%)
AUC (95% CI)	0.966 (0.94–0.98)
PPA (95% CI)	98.7% (95.2–99.7%)
NPA (95% CI)	96.1% (93–97.8%)
PLR (95% CI)	115.12 (28.94–457.96)
NLR (95% CI)	0.061 (0.033–0.111)
Accuracy (95% CI)	97.08% (94.96–98.48%)

*AUC* Area under the curve, *PPA* Positive predictive agreement, *NPA* Negative predictive agreement, *PLR* Positive likelihood ratio, *NLR* Negative likelihood ratio, *Cl* confidence interval.

DIAGNOVIR detected 39/39 (100%) of the asymptomatic patients (Table 3). Ct values of these samples were as follows (positive/total): 22/39 (56.4%), 12/39 (30.8%), and 5/39 (12.8%) for the ct values < 26, 26–30, and 31–35, respectively. The sensitivity and specificity of DIAGNOVIR in the asymptomatic sub-group were 100% (95% CI 90.98–100%) and 96.97% (95% CI 84.24–99.92%), respectively. In our study group, Ct values for the symptomatic patients (26.51 ± 5.30) were significantly lower than the asymptomatic (23.39 ± 7.16) patients (Mean ± SD; p < 0.05). The diagnostic performance of DIAGNOVIR for asymptomatic patients is summarized in Table 4.

**Table 3:** SARS-CoV-2 RT-PCR and DIAGNOVIR test results for asymptomatic patients.

DIAGNOVIR performance	PCR/Negative n (%)	PCR/ Positive samples stratified through Ct values n (%)		
		< 26	26–30	31–35
DIAGNOVIR/Negative	32 (97)	0	0	0
DIAGNOVIR/Positive	1 (3)	22 (100)	12 (100)	5 (100)
Total	33 (100)	22 (100)	12 (100)	5 (100)

**Table 4:** SARS-CoV-2 RT-PCR and DIAGNOVIR test results for asymptomatic patients.

DIAGNOVIR performance	All asymptomatic patients
Sensitivity (95% CI)	100% (90.98–100%)
Specificity (95% CI)	96.97% (84.24–99.92%)
AUC (95% CI)	0.985 (0.923–1.00)
PPA (95% CI)	97.5% (84.99–99.63%)
NPA (95% CI)	100%
PLR (95% CI)	33
NLR (95% CI)	0
Accuracy (95% CI)	%98.6 (%92.5-%99.97)

*AUC* Area under the curve, *PPA* Positive predictive agreement, *NPA* Negative predictive agreement, *PLR* Positive likelihood ratio, *NLR* Negative likelihood ratio, *Cl* confidence interval.

### Daily viral kinetics of SARS-CoV-2 from six patients

We performed the viral kinetics by serial qRT-PCR and DIAGNOVIR antigen test of nasopharyngeal swabs specimens from six patients. A total of 24 samples were collected from these patients. We classified the patient's qRT-PCR and DIAGNOVIR antigen test results from the date of symptom. All patients’ symptoms started two days before the first qRT-PCR and DIAGNOVIR antigen tests and average Ct values for 1st, 3rd, 5th, and 7th day were calculated as 26,33 ± 5.92, 31.16 ± 4.49, 32.20 ± 3.70 and 32.66 ± 3.21, respectively. As shown in Table 5, all qRT-PCR and DIAGNOVIR antigen test results were positive on the first testing day but DIAGNOVIR antigen test results started turning negative after the 5th day of the symptom onset.

**Table 5:** Daily Viral Kinetics of SARS-CoV-2 from six patients.

Patient no	1st day			3rd day			5th day			7th day		
	PCR	Ct value	DIAGNOVIR	PCR	Ct value	DIAGNOVIR	PCR	Ct value	DIAGNOVIR	PCR	Ct value	DIAGNOVIR
1	P	33	P	P	35	P	N	–	N	N	–	N
2	P	31	P	P	35	P	P	35	N	N	–	N
3	P	28	P	P	24	P	P	26	P	P	29	P
4	P	22	P	P	32	P	P	33	P	P	35	N
5	P	27	P	P	32	P	P	32	P	P	34	N
6	P	17	P	P	28	N	P	35	N	N	–	N
Total (P/N)	6/0	26.33 ± 5.92	6/0	6/0	31.16 ± 4.49	5/1	5/1	32.20 ± 3.70	3/3	3/3	32.67 ± 3.21	1/5

Total values are given as Mean ± SD.

*P* Positive, *N* Negative.

### DIAGNOVIR COVID-19 rapid antigen test sensitivity and limit of detection (LoD)

To analyze the sensitivity of Diagnovir, Ct values and viral load calculated samples collected from 19 patients were used. Samples, with various concentrations of the SARS-CoV-2 virus, were analyzed with antigen testing. SARS-CoV-2 virus was spiked into the collection buffer to get a final concentration ranging from 1.8 × 10^6^/mL to 0.25 × 10^4^/mL. We determined that the DIAGNOVIR COVID-19 Rapid Antigen Test was highly accurate when the viral load was > 0.9 × 10^4^ copies/ml. The DIAGNOVIR determined COVID-19-positive samples with 100% concordance when compared with qRT-PCR if the viral load was > 1.8 × 10^4^ copies/ml in the sample (Table 6).

**Table 6:** Determination of viral load from RT-PCR Ct value and performance of DIAGNOVIR COVID-19 Rapid Antigen Test.

Viral Content	Ct value	Viral particle/reaction	Result (positive/total)
1.8*10^6^/mL	23.00 ± 1.41	36,000/20 µL	20/20
1.8*10^5^/mL	24.33 ± 1.53	3600/20 µL	20/20
1.8*10^4^/mL	26.00 ± 2.94	360/20 µL	20/20
0.9*10^4^/mL	30.50 ± 3.61	180/20 µL	19/20
0.5*10^4^/mL	34.25 ± 1.26	100/20 µL	8/20
0.25*10^4^/mL	36.63 ± 3.50	50/20 µL	0/20

Ct values are given as Mean ± SD.

The DIAGNOVIR COVID-19 Rapid Antigen Test LoD was determined by testing ten-fold dilutions of chemically (Beta-propioactone, Sigma) inactivated SARS-CoV-2 virus isolated from SARS-CoV-2 RT-PCR positive nasopharyngeal swab of a patient and confirming the lowest detectable concentration of SARS-CoV-2 at which 95% of the analyzes resulted in a positive test. The DIAGNOVIR COVID-19 Rapid Antigen Test’s LoD was calculated as 8 × 10^2^, 6.81 × 10^1.5^, 3.2 × 10^1.5^, 1 × 10^3^, 1 × 10^3.5^ TCID50/mL for Wuhan, alpha (B1.1.7), beta (B.1.351), delta (B.1.617.2) and omicron (B.1.1.529) variants, respectively (Table 7). The positive samples used for LoD were confirmed by using the q-RT-PCR kits.

**Table 7:** DIAGNOVIR COVID-19 Rapid Antigen Test Limit of Detection (LoD).

2019-nCoV Strain Tested : MT675956						
Variant: Wuhan						
Stock Titer: 8*10^6^ TCID50/mL						
Diluation	1/10	1/100	1/1000	1/10,000	1/30,000	1/100,000
Concentration	8*10^5^	8*10^4^	8*10^3^	8*10^2^	8*10^1.5^	8*10^1^
Call rate	100/% (35/35)	100/% (35/35)	100/% (35/35)	100/% (35/35)	94.3/% (33/35)	0/% (0/35)
Limit of Detection (LoD) per virus strain: 8 X 10^2^ TCID50/mL						

2019-nCoV Strain Tested : EPI_ISL_1661919						
Variant: Alpha						
Stock Titer:6.81*10^6^ TCID50/mL						
Diluation	1/10	1/100	1/1000	1/10,000	1/30,000	1/100,000
Concentration	6.81*10^5^	6.81*10^4^	6.81*10^3^	6.81*10^2^	6.81*10^1.5^	6.81*10^1^
Call rate	100/% (35/35)	100/% (35/35)	100/% (35/35)	100/% (35/35)	97.1/% (34/35)	0/% (0/35)
Limit of Detection (LoD) per virus strain: 6.81 X 10^1.5^ TCID50/mL						

2019-nCoV Strain Tested : EPI_ISL_10335002						
Variant: Beta						
Stock Titer:3.2*10^5^ TCID50/mL						
Diluation	1/10	1/100	1/1000	1/3000	1/10,000	
Concentration	3.2*10^4^	3.2*10^3^	3.2*10^2^	3.2*10^1.5^	3.2*10^1^	
Call rate	100/% (35/35)	100/% (35/35)	100/% (35/35)	97.1/% (34/35)	0/% (0/35)	
Limit of Detection (LoD) per virus strain: 3.2 X 10^1.5^ TCID50/mL						

2019-nCoV Strain Tested : EPI_ISL_9988836						
Variant: Delta						
Stock Titer:1*10^5^ TCID50/mL						
Diluation	1/10	1/100	1/300	1/1000	1/3000	
Concentration	1*10^4^	1*10^3.5^	1*10^3^	1*10^2^	1*10^1.5^	
Call rate	100/% (35/35)	100/% (35/35)	100/% (35/35)	91.4/% (32/35)	0/% (0/35)	
Limit of Detection (LoD) per virus strain: 1X10^3^ TCID50/mL						

2019-nCoV Strain Tested : EPI_ISL_9991397						
Variant: Omicron						
Titer:1*10^6^ TCID50/mL						
Diluation	1/10	1/100	1/300	1/1000	1/3000	
Concentration	1*10^5^	1*10^4^	1*10^3.5^	1*10^3^	1*10^2.5^	
Call rate	100/% (35/35)	100/% (35/35)	100/% (35/35)	91.4/% (32/35)	0/% (0/35)	
Limit of Detection (LoD) per virus strain: 1X10^3.5^ TCID50/mL						

### Cross-reactivity for DIAGNOVIR COVID-19 rapid antigen test

No cross-reactivity was observed for the Human Coronavirus (hCoV229E) and Human Rhinovirus 1B, Respiratory syncytial Virus (RSV), Parainfluenza (type 3), and Influenza A (AH3, with the exception of SARS-CoV2 when tested with the DIAGNOVIR. Each virus spiked into a negative collection buffer for testing (Table 8).

**Table 8:** Cross-reactivity of DIAGNOVIR with clinical samples without SARS-CoV-2.

	Number of samples	Positive/Total	Cross reactivity (Yes/No)
Pathogen			
hCoV229E	10	0/10	No
HRV	10	0/10	No
RSV	10	0/10	No
Parainfluenza (type 3)	10	0/10	No
Influenza A (AH3)	10	0/10	No

*hCoV229E* human coronavirus, *HRV* Human Rhinovirus 1B, *RSV* respiratory syncytial virus.

## Discussion

Antigen-based immunoassay tests for SARS-CoV-2 can detect the presence of specific viral antigens including spike or nucleocapsid protein in fluorescent-based, cartridge-based, or lateral-flow format. Antigen tests are currently consented to be performed on nasal, oropharyngeal, and/or nasopharyngeal specimens placed directly into the specific extraction buffer or reagent. The results in antigen tests are generally obtained within half an hour and their reliability is comparable to the RT-PCR Tests for high viral load samples^12,13^. Most of the antigen tests are in lateral flow format and performed by self-test. Within these 2 years, lateral flow antigen tests were well evaluated for the sensitivity that they were less sensitive than PCR.

The development of better and faster testing against viral infections, together with vaccination, is the key to containing infectious diseases that seriously threaten public health. As shown in this study, DIAGNOVIR diagnostic antigen test satisfies the prerequisites for such a critical mission.

European Union/European Economic Area (EU/EEA) the Member States and US Food and Drug Administration (FDA) have agreed on the use of the approved rapid antigen detection tests (RADTs). The performance of the RADTs is an important criterion for the selection. According to WHO, the minimum performance criteria of the RADTs must have a ≥ 80% sensitivity and ≥ 97% specificity. RADTs are also approved by the European Centre for Disease Prevention and Control (ECDC)^14,15^.

ECDC has accepted the minimal criteria with a performance closer to RT-PCR, which is ≥ 90% sensitivity and > 98% specificity, as also recommended by EU Health Security Committee (HSC)^15^. Since the beginning of the pandemic, RT-PCR has routinely been used and remained the gold standard to confirm the diagnosis^16–18^. However, the clinical sensitivity of the RT-PCR can decrease up to 40%^19–21^. Therefore, RADTs became popular and are used widely in Europe since December 2020, as they are easy to access and use, give rapid results, and are cost-effective compared to RT-PCR^14,15^.

In addition to performance and promptness, there are other advantages of RADTs such as scalability, logistic, material, and human resources. RADTs do not require a transfer of the samples, highly equipped laboratories, and dedicated and trained laboratory professionals.

DIAGNOVIR is a new point-of-care antigen test platform that can rapidly detect the presence of SARS-CoV-2 using chips specialized for FRET with significant reliability. It was also successful in detecting asymptomatic cases of COVID-19 infection.

In our study, we evaluated the performance of the DIAGNOVIR and compared the results with those of the qRT-PCR test. DIAGNOVIR can process and give the results in 60 s, which greatly shortens the test duration. The overall sensitivity and the specificity of the device were 94% and 99.2%, respectively for the 411 symptomatic patients which are higher than the minimum criteria determined by WHO^2,14,15^.

Positive percent agreement (PPA) and Negative percent agreement (NPA) of all NAAT and antigen tests vary depending on the pre-testing probability^22^. DIAGNOVIR rapid test was also found to have a PPA and NPA of 99% and 96%, respectively among symptomatic patients.

It was shown that RADTs were most successful to identify RT-PCR-positive asymptomatic or symptomatic patients with higher viral load (i.e. Ct values < 26)^23^. However, DAIGNOVIR performance was high even with a low viral load. While the sensitivity of the RADTs for samples with Ct values < 26 was 92.5%, 98.9%, 97.4% and 95% for the Lumipulse, Panbio, SD Biosensor, and Roche, it was 99.2 for the DIAGNOVIR^24–26^. However, Lumipulse, Panbio, and SD Biosensor had a higher sensitivity for the Ct values lower than 18 or 20, respectively^24,26^. Sensitivities of the RATs decreased relatively with higher Ct values. The sensitivity of the Lumipulse, Panbio, SD Biosensor, and Roche for samples with Ct values between 26 to 30 was 92.5%, 41.3%, 52.2%, and 44.8% respectively, and lower than 50%, if the Ct values between 30–35, while it was 90.1 and 89.5 for the DIAGNOVIR, respectively^24–26^. Specificity of the DIAGNOVIR was 99.2, while it was 99%, 100%, 97.3%, and 96% for the Lumipulse, Panbio, SD Biosensor, and Roche, respectively, for Ct values up to 35^24–26^.

Patients with no symptoms at the screening point were defined as having asymptomatic infections, which included infected people who have not yet developed symptoms but go on to develop symptoms later (presymptomatic infections), and those who are infected but never develop any symptoms (true asymptomatic or covert infections). It was shown that viral load was directly associated with disease severity and it is leading to severe forms of the infection^27^. However, our observations showed that Ct values for the patient with no symptoms were significantly lower compared to those with the symptoms. It might be related to our study group, which included more presymptomatic patients as it was found that Ct values were similar between asymptomatic and presymptomatic patients^27,28^. Besides, some studies have reported no correlation between Ct value and clinical severity^29,30^.

The high rate of asymptomatic people in the community is responsible for the potentially significant uncontrolled transmission of COVID-19. The studies showed that the proportion of asymptomatic infections ranged from about 1–80%^31,32^. Scale-up of testing can prevent the spread and tackle the pandemic. As shown in Tables 3 and 4, the sensitivity of the DIAGNOVIR rapid test was found to be 100% among asymptomatic patients, and it was 97.5%, 48.1%, 48.1% and 71.43% for the Lumipulse, Panbio, SD Biosensor, and Roche, respectively^33–36^. Some studies suggested that the peak of transmissibility is at or even before the time of symptom onset. Both asymptomatic and symptomatic patients have a certain frame of viral shedding like 7–8 days which suggests that patients without symptoms can still transmit the virus^37,38^. Although transmission of the virus depends on multiple factors and there is uncertainty regarding the asymptomatic transmission; joint BIA, HIS, IPS, and RCPath guidance reports that presymptomatic transmission is confirmed and asymptomatic transmission is probable^39^. DIAGNOVIR is a device dependent antigen test device, nevertheless it is still convenient to detect probable asymptomatic patients who may cause the spread of the disease. Particularly the ability to detect asymptomatic infections with an excellent percentage makes it a convenient tool in the world’s current struggle against the pandemic.

Serial or frequent testing of the target population regardless of their signs or symptoms combined with other prevention strategies is a key component to reducing the transmission of the virus^40,41^. There is considerable confusion around the infectious period from onset to recovery. It is reported that the incubation period for infection with SARS‑CoV‑2, which is required to determine the appropriate duration of quarantine, may range from 2 to 14 days^42,43^. It is important to perform serial testing during this period. However, it has been shown that prolonged detection of SARS‑CoV‑2 RNA by RT-PCR is common and it can reach up to 12 weeks after symptom onset, without indicating contagiousness^44^. Our results show that DIAGNOVIR can detect SARS‑CoV‑2 with 100% sensitivity up to 5 days after symptom onset. DIAGNOVIR antigen test results started turning negative after the 5th day of the onset of symptoms, which is related to the low viral load.

The limit of detection (LoD) is a measure of analytic sensitivity and is generally considered the lowest detectable concentration of SARS-CoV-2 in ≥ 95% of repeated measurements analyzed resulting in a positive test for molecular diagnostic assays^45,46^. It has been shown that the viral load is associated with symptom onset, although a high viral load was observed in asymptomatic patients. Even though the viral load is decreasing day by day after the symptom onset, patients can be potentially contagious with a low viral load. Thus, RADTs show the highest sensitivity in identifying the low viral load and are the most reliable. LoD for DAIGNOVIR was calculated as > 0.9 × 10^4^ copies/ml at a level of confidence of 95% symptomatic patients with disease onset ≤ 5 days to identify individuals.

Diagnostic testing for SARS-CoV-2 is challenged due to several mutations on spike and nucleocapsid proteins and the results of the antigen tests are affected by viral mutations^47^. To understand the effect of mutations on DIAGNOVIR’s test performance, we performed the LoD studies for each SARS‑CoV‑2 variant. We observed that DIAGNOVIR Rapid Antigen Test’s LoD was not significantly affected by the SARS‑CoV‑2 variants and the mean LoD was calculated as 1.05 × 10^3^ TCID50/mL, with a range of 3.2 10^1.5^–1 × 10^3.5^ TCID50/mL. It was 1.0 × 10^5^, 1.5 × 10^1.8^, 5 × 10^2^, and 3.1 × 10^2.2^ for the Lumipulse, Panbio, SD Biosensor, and Roche, respectively^24,48–50^.

One of the advantages of antigen-based assays is timing. They provide immediate results compared to PCR-based tests. Most RADTs had a fast turnaround time of 15–30 min. However, DIAGNOVIR has the fastest turnaround time based on FRET technology (< 2 min).

Point-of-care testing for infectious diseases can depend on various strategies, with each one having its pros and cons. In the case of DIAGNOVIR, the main disadvantages appear to be the requirement for a specialized device and single-use experiment kits, which are also necessary components of PCR tests. These may increase the overall cost of the DIAGNOVIR test system; yet the difference with alternative methods, such as lateral flow tests could also be negligible when volume production is considered as the single-use experiment kit of DIAGNOVIR are cheaper than equivalent antigen test kits on the market. Besides, most of the consumables such as swabs are shared among all test types. Taken together, DIAGNOVIR ultra-rapid diagnostic test presents a high analytical performance in terms of sensitivity and specificity even with a low viral load, including for the asymptomatic and symptomatic patients compared to other most studied rapid antigen test approaches.

Considering the probe antibodies can always be updated with respect to the upcoming mutations, without compromising the FRET-based detection strategy, DIAGNOVIR finds itself a significant role in infection detection including viral and bacterial infections, and all other diagnostic tests that involve antibody-antigen in the future is highly likely.

## Conclusions

RADTs with higher sensitivity and specificity can significantly contribute to COVID-19 testing capacity, reduce transmission with early detection, lower testing costs, decrease turnover time, and in this wise control the pandemic. This study demonstrated that DIAGNOVIR is highly effective in both asymptomatic and symptomatic populations and it can detect all SARS-CoV-2 variants in just seconds with high sensitivity and specificity compared to RT-PCR and can be used as a testing device. The FRET-based detection enables a fast and accurate method for identification of the infection, which is a powerful tool in the battle against the pandemic.

## Limitations

As authors, we are aware of the following limitations of the study: We performed the cross-reactivity study using a set of viruses available at the time of the study; therefore, a more complete cross-reactivity study can be performed in the future. Nevertheless, the data collected shows no significant cross-reactivity. Since the investigated test is a point-of-care system, we have not investigated the viral transfer medium conditions, and we have not performed a freezing-rethawing study at − 80 °C. The testing of the efficiency in different samples such as sputum or saliva is also a topic for further research, which we believe will be a valuable study for the evaluation of FRET-based SARS-CoV-2 testing. Another limitation that can be considered is the sample size and the uneven distribution of the subsamples. For instance, the distribution of COVID-19-positive patients with different Ct counts does not have an approximate ratio of 1:1:1 (Table 1). Yet, the values are not extremely different from this ratio, either. A larger-scale study may yield a more comprehensive output. Nevertheless, the statistical significance of the results presented here is still valid.

## Material and methods

### Patients and samples

The study was conducted at Ankara University School of Medicine, Department of Infectious Diseases and Clinical Microbiology and all methods were performed in accordance with the relevant guidelines and regulations. Adult patients who either applied to the COVID-19 outpatient clinic or followed in COVID-19 inpatient clinics were eligible for study participation. Participants were enrolled upon signing informed consent. For outpatients, two nasopharyngeal swab samples were taken simultaneously; one for SARS-CoV-2 RT-PCR and one for DIAGNOVIR testing. PCR was chosen as the gold standard to define COVID-19 and all DIAGNOVIR results were compared with the RT-PCR test results. The PCR-positive patients were divided into 3 groups according to the Ct values in the study as higher, moderate, or lower virus load (< 26, 26–31, 31–35), and the cut-off Ct value was determined as 35 according to the manufacturer^35,51^. For inpatients, to evaluate the diagnostic performance of DIAGNOVIR at varying viral loads and days of illness, four samples were taken on days 1, 3, 5, and 7 of hospital admission; one nasopharyngeal swab for SARS-CoV-2 RT-PCR, one for DIAGNOVIR. All samples were collected with polyester swabs were placed into viral nucleic acid lysis buffer (vNAT, Biospeedy, Turkey) or sample collection buffer for RT-PCR analysis and DIAGNOVIR testing, respectively. vNAT and sample collection buffers were immediately stored at 4 °C until nucleic acid extraction RT-PCR testing was performed within 2–4 h.

### DIAGNOVIR antigen test

Samples for DIAGNOVIR were collected on-site using the swabs provided by the manufacturer (Felisya Biyomedikal, Turkey) and placed into a sample collection buffer, and kept at room temperature. Antigen tests were performed within 15 min after the specimen collection and results were recorded as negative or positive in a time span period of one minute. DIAGNOVIR is an immune-fluorescence analysis based on FRET measurements. In summary, the system measures the FRET signal changes. The excitation is set to activate the donor fluorophore only. If the viral protein is present in the solution, it will bind to the antibodies located on the surface of the chip, bringing donor and acceptor fluorophores in a close vicinity adequate to observe the FRET, which will cause an increase in the fluorescence intensity in the longer wavelength of the FRET pair. These fluorescence changes will be detected by the fluorescence detector in the system. The screen embedded in the system shows negative or positive results depending on the presence of viral particles in the solution.

### Viral nucleic acid extraction and RT-qPCR

Total nucleic acid was isolated from nasopharyngeal swabs using the EZ1 virus mini kit (Qiagen, USA) on the EZ1 Advanced XL platform (Qiagen, USA) according to the manufacturer’s instructions. For RT-PCR analysis, the SARS-CoV-2 N1 + N2 Assay Kit (Qiagen, USA) was used according to the manufacturer's instructions and amplification was performed on the Rotor-Gene Q 5Plex HRM (Qiagen, Malaysia) platform with the following cycling conditions: 50 °C (10 min) for reverse transcription, 95 °C (2 min) for inactivation, 40 cycles of 95 °C (5 s) for denaturation and 58 °C (30 s) for combined annealing/extension. A threshold cycle (Ct) value was assessed for each PCR reaction and the amplification curve was visually evaluated and results were recorded.

### SARS-CoV-2 virus isolation

To test the performance of DIAGNOVIR for different variants, alpha, beta, delta, and omicron variants isolated by cell culture were used, in addition to patient samples. SARS-CoV-2 Wuhan virus and variants (alpha, beta, delta, and omicron) were isolated from the SARS-CoV-2 PCR-positive nasopharyngeal specimen. SARS-CoV-2 was cultured on Vero E6 cells (ATCC CRL-1586) with DMEM high glucose (Gibco, 41,966–029) supplemented with % 5 Fetal Bovine Serum (Gibco, 10,500,064), %1 Penicillin–Streptomycin and Amphotericin B (Sigma, A2942). Cytopathic effect (CPE) was monitored for 5–7 days. Fifty-percent tissue culture infective dose (TCID50) assay was performed in 96 well plates and TCID50 was calculated according to Spearman&Karber and Reed&Muench algorithms. Viral plaque assays were assessed for each variant and plaques were counted with the naked eye and the Celigo Image cytometer (Nexcelom, Celigo Image Cytometer 200-BFFL-5C) to determine plaque-forming units (PFU/ml). The isolates’ GeneBank accession numbers and plaque-forming unit numbers are described in Table 7.

### Limit of detection (LoD) assay and evaluation of analytical performance

Samples were collected from 6 hospitalized patients diagnosed with COVID-19 positive for the analytical performance of DIAGNOVIR and daily viral kinetics of SARS-CoV-2. Ct values and the virus concentration were determined by comparing the results obtained by quantitative RT-PCR (qRT-PCR) analysis. For this purpose, a qRT-PCR test kit (Genesig 2019-nCOV Advanced kit; Primerdesign, England) was used to detect the viral load in the patient samples according to the manufacturer’s protocol. Total nucleic acid was extracted from hospitalized patients’ nasopharyngeal swabs by using the EZ1 virus mini kit (Qiagen, USA) on the EZ1 Advanced XL platform (Qiagen, USA). For quantification, 2 × 10^5^ copy/µl SARS-CoV-2 RNA supplied by the kit was used to prepare serial tenfold dilution standards from 2 × 10^5^ copy/µl to 2 × 10^1^ copy/µl. qRT-PCR assays were conducted on the Rotor-Gene Q 5Plex HRM (Qiagen, Malaysia) platform with the following cycling conditions: 55 °C (10 min) for reverse transcription, 95 °C (2 min) for inactivation, 50 cycles of 95 °C (10 s) for denaturation and 60 °C (60 s) for combined annealing/extension. Evaluation of the results was conducted according to the manufacturer’s protocol. Endogenous internal control amplifications were observed from the yellow channel, exogenous control (sample RNA added into human extracted RNA) amplifications were observed from the red channel, viral target (RdRp gene) and standard amplifications were observed from the green channel. A threshold cycle (Ct) value was assessed for each qRT-PCR reaction and viral loads were assessed by using the amplification curves of the standards. Under optimal PCR conditions, Genesig 2019-nCoV detection kits have very high priming efficiencies (> 90%) and can detect less than 100 copies of the target template.

Daily viral kinetics of SARS-CoV-2 were performed from 24 nasopharyngeal samples collected from the 6 patients every 2 days. qRT-PCR and DIAGNOVIR antigen test results were analyzed and recorded from the date of symptom.

For analytical performance, positive samples collected from 3 hospitalized patients with an average Ct value of 22 were mixed and spiked into collection buffer to give a final viral concentration of 1.8 × 10^6^ /mL, 1.8 × 10^5^ /mL, 1.8 × 10^4^ /mL, 0.9 × 104 /mL, 0.5 × 10^4^ /mL, and 0.25 × 10^4^ /mL with corresponding Ct values, obtained by the qRT-PCR and RT-PCR analysis for each dilution, respectively. 20 µL of the spiked collection buffer was applied per test kit. The average viral concentration per reaction (Viral Particle/reaction in 20µL) was 50, 100, 180, 360, 3600 and 36,000, respectively. The DIAGNOVIR test was performed 20 times for each dilution as shown in Table 6.

LoD of the DIAGNOVIR COVID-19 Rapid Antigen Test was evaluated by testing chemically (Beta-propioactone, Sigma) inactivated SARS-CoV-2 virus isolated from the RT-PCR positive nasopharyngeal swab of patients. Testing dilutions of the viruses were assessed to determine the LoD of the DIAGNOVIR against Wuhan, alpha, beta, delta, and omicron variants (one strain per each variant). The DIAGNOVIR test was performed 20 times for each variant as shown in Table 7.

### Cross-reactivity

Human Coronavirus (hCoV229E) and Human Rhinovirus 1B, Respiratory syncytial Virus (RSV), Parainfluenza (type 3), and Influenza A (AH3) were tested for cross-reactivity. For Human Coronavirus (hCoV229E) and Human Rhinovirus 1B, the culture stocks were used. Briefly, hCoV229E (ATCC VR-740) virus and human Rhinovirus 1B (ATCC VR-1645) were purchased from the ATCC culture collection. hCoV-299E was cultured on MRC5 cells (ATCC CCL-171) and Human rhinovirus 1B was cultured on Hela cells. The TCID50 of Human Coronavirus 229E and Human Rhinovirus 1B were 9.2 × 10^3^ and 6.81 × 10^5^, respectively. Respiratory syncytial Virus (RSV), Parainfluenza (type 3), and Influenza A (AH3) cross-reactivity were tested with nasopharyngeal specimens which were found to be positive by RT-PCR.

### Statistical analysis

MedCalc^®^ Statistical Software version 19.7.2 (MedCalc Software Ltd, Ostend, Belgium; https://www.medcalc.org; 2021) program was used for statistical analysis. While continuous variables were described in mean (± standard deviation) and median (minimum–maximum), categorical variables were identified with frequency (n) and percent (%). The Chi-square test was chosen for the comparison of the ratios. To evaluate the performance of the new diagnostic test, sensitivity, specificity, positive and negative predictive values, positive and negative likelihood ratios, and accuracy were interpreted with a 95% confidence interval. ROC analysis was used to determine the diagnostic performance of DIAGNOVIR according to viral load. P < 0.05 value was assigned to indicate statistical significance.

### Ethical approval

The study was approved by the Ankara University Medical Faculty Ethics Committee and the Turkish Ministry of Health, Medical Device Notified Body and Clinical Research Department with the decision numbers 06-243-21 and E-381032, respectively.

## Supplementary Information

Supplementary Table 1.

## Data Availability

The datasets supporting the conclusions of this article is(are) included within the article.
