# Supplementary material for: Clinical evaluation of DIAGNOVIR SARS-CoV-2 ultra-rapid antigen test performance compared to PCR-based testing
Source: Sci Rep. 2023 Mar 17;13:4438. doi: 10.1038/s41598-023-31177-8 (PMC10021059; doi:10.1038/s41598-023-31177-8)
Supplement: Supplementary file 1 — Supplementary Table 1. [file 41598_2023_31177_MOESM1_ESM.docx]

**Supplementary material 1. Ct values of SARS-CoV-2 Rt-qPCR positive samples and DIAGNOVIR test results**

| **No** | **Ct** | **DIAGNOVIR result** | **No** | **Ct** | **DIAGNOVIR result** | **No** | **Ct** | **DIAGNOVIR result** | **No** | **Ct** | **DIAGNOVIR result** |
| --- | --- | --- | --- | --- | --- | --- | --- | --- | --- | --- | --- |
| **1** | 5 | Positive | **43** | 23 | Positive | **85** | 27 | Positive | **127** | 30 | Positive |
| **2** | 7 | Positive | **44** | 23 | Positive | **86** | 27 | Positive | **128** | 30 | Positive |
| **3** | 8 | Positive | **45** | 23 | Positive | **87** | 27 | Positive | **129** | 31 | Negative |
| **4** | 9 | Positive | **46** | 23 | Positive | **88** | 27 | Positive | **130** | 31 | Negative |
| **5** | 15 | Positive | **47** | 23 | Positive | **89** | 27 | Positive | **131** | 31 | Positive |
| **6** | 15 | Positive | **48** | 23 | Positive | **90** | 27 | Positive | **132** | 31 | Positive |
| **7** | 15 | Positive | **49** | 23 | Positive | **91** | 27 | Positive | **133** | 31 | Positive |
| **8** | 15 | Positive | **50** | 23 | Positive | **92** | 27 | Positive | **134** | 31 | Positive |
| **9** | 16 | Positive | **51** | 23 | Positive | **93** | 27 | Positive | **135** | 31 | Positive |
| **10** | 16 | Positive | **52** | 24 | Positive | **94** | 27 | Positive | **136** | 31 | Positive |
| **11** | 16 | Positive | **53** | 24 | Positive | **95** | 27 | Positive | **137** | 31 | Positive |
| **12** | 16 | Positive | **54** | 24 | Positive | **96** | 28 | Positive | **138** | 31 | Positive |
| **13** | 17 | Positive | **55** | 24 | Positive | **97** | 28 | Positive | **139** | 31 | Positive |
| **14** | 17 | Positive | **56** | 24 | Positive | **98** | 28 | Positive | **140** | 31 | Positive |
| **15** | 17 | Positive | **57** | 24 | Positive | **99** | 28 | Positive | **141** | 31 | Positive |
| **16** | 18 | Positive | **58** | 24 | Positive | **100** | 28 | Positive | **142** | 31 | Positive |
| **17** | 18 | Positive | **59** | 24 | Positive | **101** | 28 | Positive | **143** | 32 | Negative |
| **18** | 18 | Positive | **60** | 24 | Positive | **102** | 28 | Positive | **144** | 32 | Positive |
| **19** | 19 | Positive | **61** | 24 | Positive | **103** | 28 | Positive | **145** | 32 | Positive |
| **20** | 19 | Positive | **62** | 24 | Positive | **104** | 28 | Positive | **146** | 32 | Positive |
| **21** | 19 | Positive | **63** | 25 | Positive | **105** | 28 | Positive | **147** | 32 | Positive |
| **22** | 19 | Positive | **64** | 25 | Positive | **106** | 28 | Positive | **148** | 32 | Positive |
| **23** | 19 | Positive | **65** | 25 | Positive | **107** | 28 | Positive | **149** | 32 | Positive |
| **24** | 20 | Positive | **66** | 25 | Positive | **108** | 29 | Negative | **150** | 32 | Positive |
| **25** | 20 | Positive | **67** | 25 | Positive | **109** | 29 | Negative | **151** | 33 | Positive |
| **26** | 20 | Positive | **68** | 25 | Positive | **110** | 29 | Positive | **152** | 33 | Positive |
| **27** | 20 | Positive | **69** | 25 | Positive | **111** | 29 | Positive | **153** | 33 | Positive |
| **28** | 20 | Positive | **70** | 25 | Positive | **112** | 29 | Positive | **154** | 33 | Positive |
| **29** | 20 | Positive | **71** | 25 | Positive | **113** | 29 | Positive | **155** | 33 | Positive |
| **30** | 21 | Positive | **72** | 25 | Positive | **114** | 29 | Positive | **156** | 34 | Positive |
| **31** | 21 | Positive | **73** | 25 | Positive | **115** | 30 | Negative | **157** | 34 | Positive |
| **32** | 21 | Positive | **74** | 26 | Negative | **116** | 30 | Negative | **158** | 34 | Positive |
| **33** | 21 | Positive | **75** | 26 | Positive | **117** | 30 | Positive | **159** | 35 | Negative |
| **34** | 22 | Negative | **76** | 26 | Positive | **118** | 30 | Positive | **160** | 35 | Positive |
| **35** | 22 | Positive | **77** | 26 | Positive | **119** | 30 | Positive | **161** | 35 | Positive |
| **36** | 22 | Positive | **78** | 26 | Positive | **120** | 30 | Positive | **162** | 35 | Positive |
| **37** | 22 | Positive | **79** | 26 | Positive | **121** | 30 | Positive | **163** | 35 | Positive |
| **38** | 22 | Positive | **80** | 26 | Positive | **122** | 30 | Positive | **164** | 35 | Positive |
| **39** | 22 | Positive | **81** | 26 | Positive | **123** | 30 | Positive | **165** | 35 | Positive |
| **40** | 22 | Positive | **82** | 26 | Positive | **124** | 30 | Positive | **166** | 35 | Positive |
| **41** | 22 | Positive | **83** | 26 | Positive | **125** | 30 | Positive |  |  |  |
| **42** | 23 | Positive | **84** | 27 | Positive | **126** | 30 | Positive |  |  |  |
